# Generative adversarial networks: multiparametric, multiregion super-resolution MRI in predicting lymph node metastasis in rectal cancer

**DOI:** 10.1186/s13244-025-02173-5

**Published:** 2026-01-05

**Authors:** Yupeng Wu, Tao Jiang, Han Liu, Shengming Shi, Apekshya Singh, Yuhang Wang, Jiayi Xie, Xiaofu Li

**Affiliations:** 1 Department of Magnetic Resonance Imaging Diagnostic, The Second Affiliated Hospital of Harbin Medical University, Harbin, China; 2 Medical Imaging Department (MRI), The Fifth Affiliated Hospital of Harbin Medical University, Daqing, China

**Keywords:** Rectal cancer, Generative adversarial network, Super resolution, Lymph node metastasis, MRI

## Abstract

**Objective:**

Development of a preoperative mesorectal lymph node metastasis (LNM) prediction model for rectal cancer (RC) based on intratumoral and multiregional peritumoral radiomics features extracted from super-resolution multiparametric MRI.

**Materials and methods:**

This multicenter study included preoperative MRI data from 243 rectal cancer patients (194 from center A, 49 from center B) with SR reconstruction and scoring. Radiomic features were extracted from tumor, peri-3mm and peri-5mm on SR-DWI and SR-T2WI images. The least absolute shrinkage and selection operator (LASSO) and the maximum relevance minimum redundancy (mRMR) were used for feature selection and dimensionality reduction. DWI_T2WI_INTRA, DWI_T2WI_IntraPeri3mm, DWI_T2WI_InterPeri5mm models were developed employing Logistic regression. Independent clinical risk factors identified through univariate and multivariate stepwise regression analyses were used to construct a clinical model. The optimal IntraPeri model integrated with clinical model design the combined model. Predictive performance was evaluated using ROC curves, calibration curves, and decision curve analysis (DCA).

**Results:**

Qualitative evaluation demonstrated superior scores for SR-T2WI across five metrics compared to original images (all *p* < 0.001). For DWI, SR images achieved significant improvements in all parameters (*p* < 0.001), except lesion conspicuity [median (IQR): 3 (1) vs. 3 (1)]. Comparative analysis revealed the DWI_T2WI_IntraPeri3mm model’s optimal predictive performance in training, validation, and test cohorts (AUCs: 0.880, 0.735, and 0.714, respectively). The AUC of the combined model, integrating radiomic (DWI_T2WI_IntraPeri3mm) model with clinical risk factors, was 0.933, 0.829, and 0.867 in each cohort, all exceeding those of the clinical and radiomic models.

**Conclusion:**

Using GANs-based 3D-SR of multi-sequence MRI, our multiregional prediction model for preoperative mesorectal LNM in RC demonstrated good diagnostic performance.

**Critical relevance statement:**

The integration of super-resolution-based tumor and peritumoral 3-mm predictive model with clinical risk factors enables performance in predicting mesorectal LNM, potentially aiding clinical therapeutic decision-making.

**Key Points:**

- How do tumor and peritumoral (3–5 mm) models-based SR images perform in predicting lymph node metastasis (LNM)?
- The DWI_T2WI_IntraPeri3mm model, when combined with clinical factors, improves diagnostic accuracy.
- Multiparametric, multiregional super-resolution (SR)-MRI radiomics models exhibit good performance for LNM.

**Graphical Abstract:**

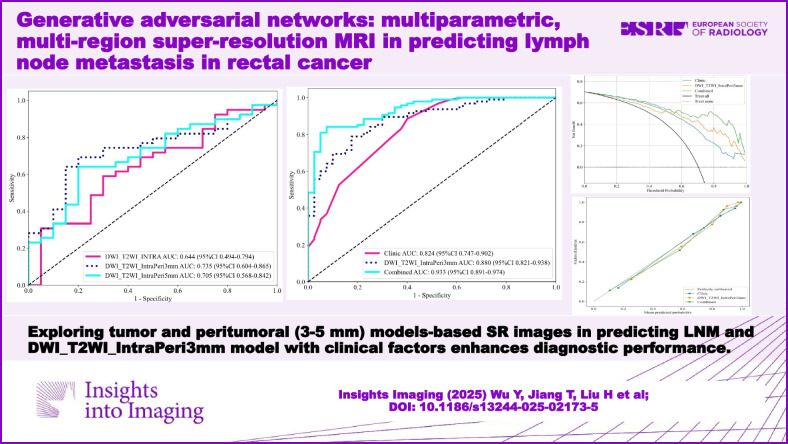

## Introduction

Colorectal cancer ranks third in global incidence and second in mortality, with male predominance [1]. Rectal cancer (RC) predominantly metastasizes to mesorectal lymph nodes (LNs), a critical prognostic indicator linked to reduced survival [2] and elevated distant metastasis rates. Studies reveal superior 5-year survival in lymph node-negative (LNN) compared with lymph node-positive (LNP) patients [3]. Precise LNP prediction guides surgical planning and neoadjuvant radiotherapy regimens. Total mesorectal excision (TME) combined with neoadjuvant therapy may reduce local recurrence and treatment-related toxicity in LNP rectal cancer [4]. MRI’s superior soft-tissue resolution [5] and unique imaging characteristics establish it as the gold-standard modality for RC TNM staging. MRI demonstrated superior diagnostic performance for LN staging compared to computed tomography (CT), with sensitivity (66% vs. 55%) and specificity (76% vs. 74%) [6]. Current diagnostic criteria for LNM per clinical guidelines [7], relying on nodal size, heterogeneous signal intensity, and border irregularity, fail to differentiate tumor-induced immune responses from true metastatic enlargement [8]. In conclusion, imaging-based LNM diagnosis remains clinically challenging [9]. As the diagnostic gold standard, postoperative pathology inherently delays treatment planning due to result latency.

Deep learning-based super-resolution reconstruction (SRR) primarily encompasses convolutional neural networks (CNNs) [10, 11] and generative adversarial networks (GANs). These frameworks overcome inherent physical limitations to synthesize perceptually enhanced SR images through systematic resolution transcension, providing an end-to-end training method to enhance image resolution. Mature SSR architectures leverage adversarial frameworks to transform low-resolution inputs into super-resolved outputs, with demonstrated success across cranial brain [12], cardiac [13], prostate [14], and abdominal [15] MRI applications in medical imaging. The basic structure can be found in Fig. S1.

Emerging evidence in RC LNM research reveals a critical oversight in conventional tumor-centric analyses that neglect peritumoral microenvironment characterization [16]. Tumor biological complexity arises not only from malignant cells but also from stromal-infiltrating components, constituting the dynamically interactive peritumoral niche that drives oncogenesis [17, 18]. Current radiogenomic findings further identify the 5 mm peritumoral margin as a histopathologically validated invasion frontier [19].

No existing studies have investigated the application of SR-enhanced multiparametric MRI for preoperative mesorectal LNM prediction. This study pioneers a radiomic framework leveraging super-resolution T2WI and DWI to quantify tumor and peritumoral heterogeneity for preoperative LNM risk stratification.

## Materials and methods

### Patients

The Institutional Review Board of the Second Affiliated Hospital of Harbin Medical University (YJSKY2024-065) and the Fifth Affiliated Hospital of Harbin Medical University (LW-2025-001) approved this retrospective multicohort study but waived the requirement for written informed consent due to the retrospective nature of this study. The study was conducted in accordance with the Declaration of Helsinki (revised 2013). Data were retrospectively collected from 375 patients with rectal adenocarcinoma diagnosed between January 2020 and May 2024 at the Second Affiliated Hospital of Harbin Medical University (Center A) and the Fifth Hospital of Harbin Medical University (Center B) between June 2020 and May 2024 after surgical interventions and subsequent pathological examinations. Strict exclusion criteria were used to maintain data integrity in this study.

Inclusion criteria included (1) primary rectal adenocarcinoma, pathologically confirmed following radical resection of RC (RRRC), with definite mesorectal LNM (for details, refer to Supplementary Text 1), (2) preoperative baseline MRI without any prior pelvic surgery; (3) acceptable original image quality for basic diagnosis, and (4) comprehensive clinical information of patients.

Exclusion criteria included (1) non-primary rectal adenocarcinoma (e.g., metastases from neuroendocrine tumors, other gastrointestinal tumors), (2) pelvic surgery, neoadjuvant radiotherapy prior to the preoperative baseline MRI, (3) extremely poor image quality unnecessary for reconstruction, and (4) lack of pathological and clinical information.

**Fig. 1:**
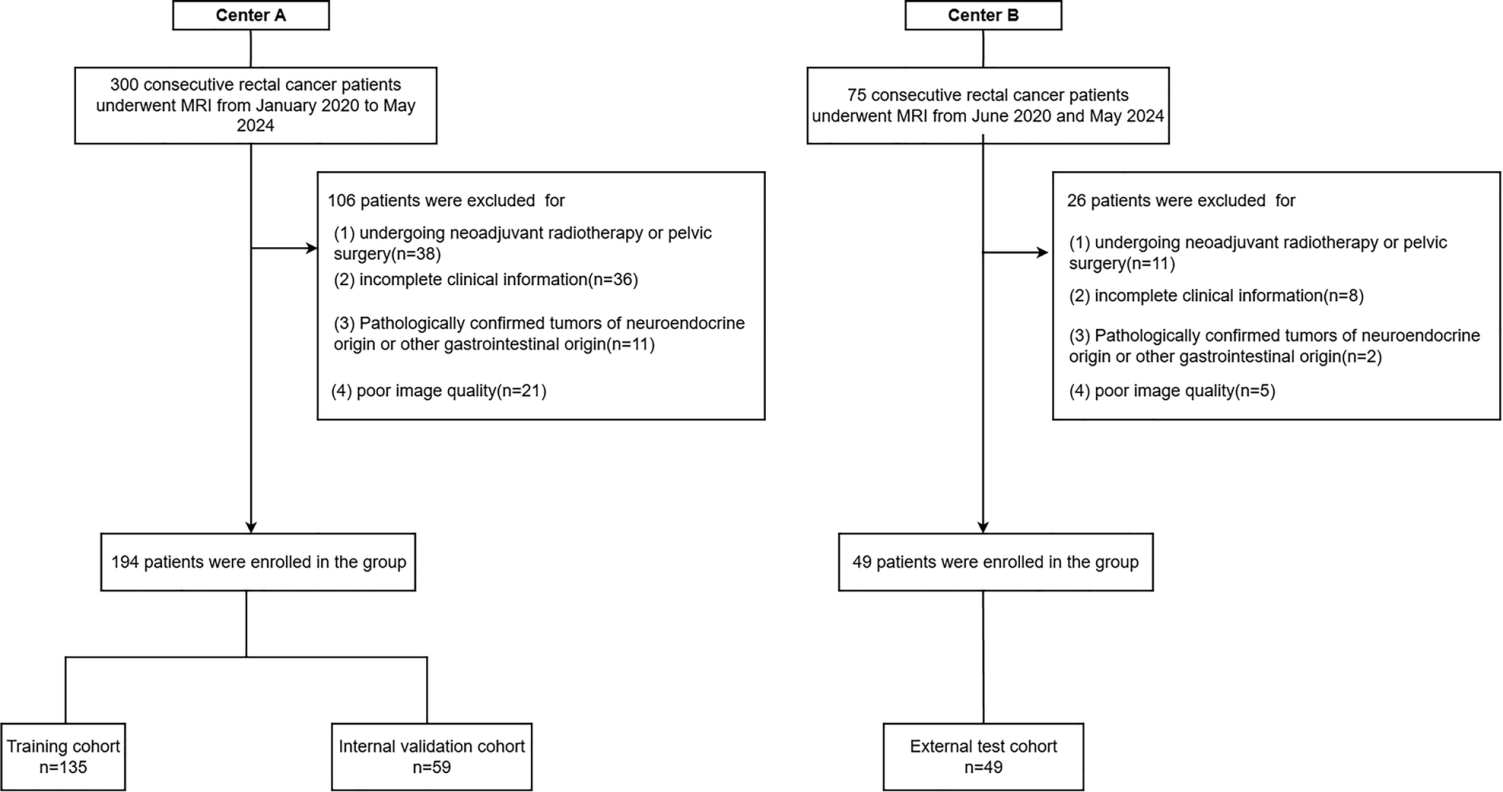
Study flowchart of the enrollment of patients

Finally, a total of 243 eligible patients were included, including 194 patients from center A and 49 patients from center B. 194 patients were randomized 7:3 into training and internal validation cohorts, and 49 patients as the external test cohort. Patient-related life information, laboratory tests and clinical information were obtained from our hospital’s electronic medical record. The flowchart of the enrollment process of patients is shown in Fig. 1.

### MRI protocol

MRI examinations were conducted using a Discovery MR750w 3.0-T MR scanner in center A and an Ingenia 3.0-T MR scanner (Philips Healthcare) in center B equipped with a phased-array coil, following a standardized protocol that captured oblique axial, sagittal, and coronal views. High-resolution oblique axial T2WI images and DWI images were analyzed in the study. Before the MRI scan, an enema was administered using water to minimize artifacts caused by intestinal contents in the images. All personal information was deidentified, and patient informed consent requirements were waived. The relevant scanning parameters are shown in Table S1.

### Clinicopathological characteristics

RRRC resection: resect tumor-containing rectal segment and peripheral connective tissue (esp. fat). Surgeons identify and retrieve palpable 1st to 3rd station mesorectal LNs (Diameter ≥ 2 mm. If < 2 mm, surrounding tissue must be submitted for examination). At least 12 LNs are collected and marked by region. All specimens were processed by professional pathologists, sectioned, stained with Hematoxylin-Eosin and observed. Mesorectal LNs (+): Clusters of malignant adenocarcinoma cells consistent with the morphology of primary rectal carcinoma (abnormal cellular structure and size, large and hyperchromatic nuclei, irregular in shape, disorganized arrangement) observed within lymphatic sinuses, lymphoid tissue, or adipose tissue. LVI (+): Clusters of carcinoma cells are identified within the lumina of lymphatic or blood vessels, lined by endothelial cells, with no red blood cell present. PNI(+): Carcinoma cells encircle nerve fascicles (involving epineurium) or invade into nerve fascicles (involving endoneurium), growing closely adjacent to nerves or within the perineural spaces.

Two experienced radiologists performed MRI evaluation: MRF(+): Tumor or metastatic lymph node is ≤ 1 mm from the mesorectal fascia (MRF), or directly penetrates and invades the MRF; EMVI(+): Tumor extends into vessels within mesorectal fat, presenting with significantly irregular thickening of peritumoral vessels, blurred tumor-vessel interface, or increased intratumoral signal intensity.

### Deep-learning super-resolution networks image reconstruction, image assessment and ROI segmentation

**Fig. 2:**
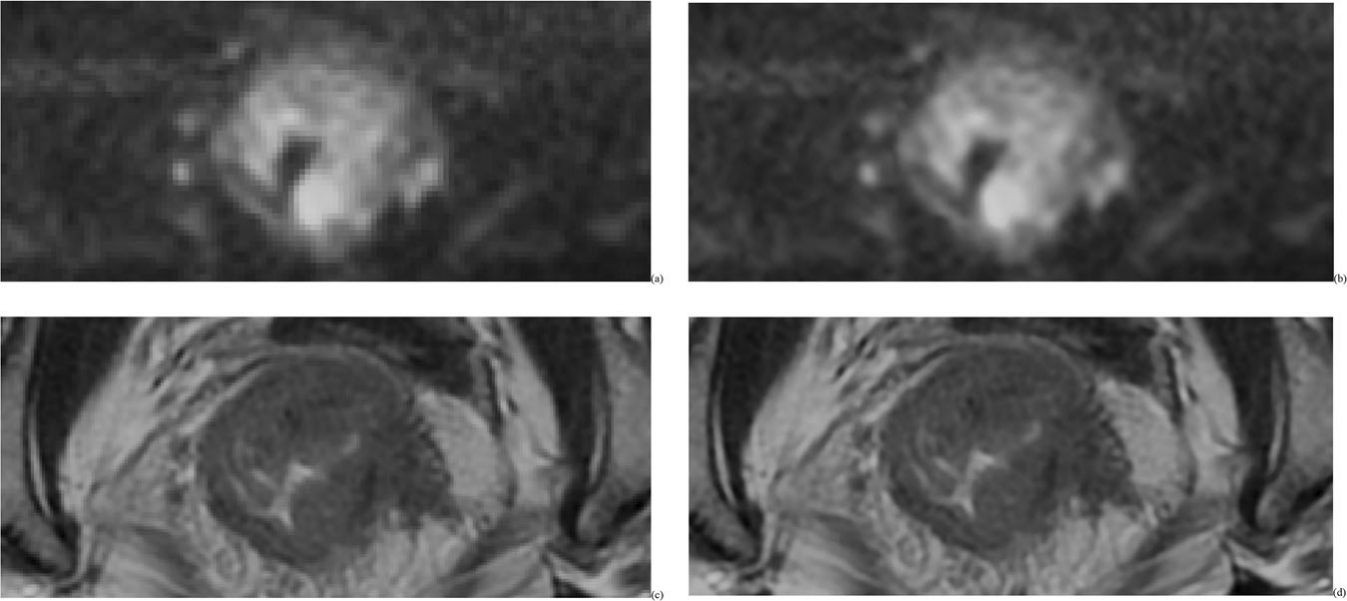
It shows the SR and ORI images: **a** ORI-DWI image; **b** the SR-DWI image; **c** the ORI-T2WI image; **d** the SR-T2WI image

In this study, pre-processed original images of oblique axial T2WI and DWI employ a GAN-based 3D medical image reconstruction technique, utilizing transfer-learning from the OneKey platform (version 3.1) to acquire SR images, as in Fig. 2. This technique fundamentally comprises two components: a generator that synthesizes realistic images and a discriminator that distinguishes between real and synthetic samples. This adversarial training process enables the GAN to learn the mapping between high-low resolution image pairs. Both entities mutually enhance their capabilities through continuous adversarial training and iterative optimization [20]. The details of the SR reconstruction process are provided in Supplementary Text 2.

Among the obtained SR images of 243 patients, a random sample of 120 patients was selected, and two experienced radiologists performed blinded evaluations and scoring of both the original images and the SR images. Based on Inoue et al’s 4-score scale [21], we designed a 4-score scale adapted to this study’s requirements (Table 1).

**Table 1:** 4-score scale suitable for the requirements of this experiment

Characteristics	Score			
	1	2	3	4
Anatomical visualization of the rectum	Poor visualization	Moderate visualization with unclear edges	Moderate visualization with clear edges	Good visualization
Lesion conspicuity	Undetectable	Barely detectable with slight contrast to the surrounding tissue	Moderate contrast with partial demarcation	Optimal contrast with complete margination
Image mosaic effect	Severe, affecting diagnosis;	Moderate, blurred image, prone to diagnostic ambiguity	Good, diagnostic quality not impaired	Almost negligible
Contrast with surrounding organs	Poor	Moderate	Good	Excellent
Overall image quality	Poor	Moderate	Good	Excellent

**Fig. 3:**
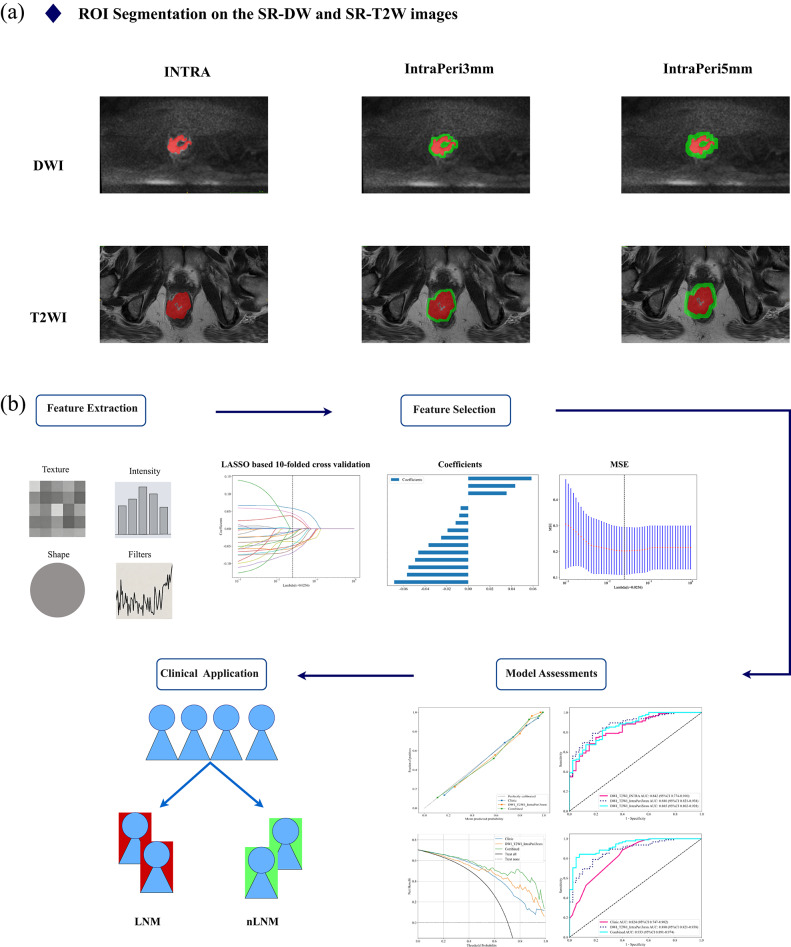
The workflow of the radiomic study. **a** The segmentation of the region of interest (ROI); **b** the rest of the workflow

Two radiologists with 10 and 15 years of experience utilized the ITK-SNAP (version 3.8.0, www.itksnap.org) software to outline a three-dimensional multilevel ROI, including the dominant slice of tumor and perilesional contiguous slices, carefully avoiding bowel contents and air. The extended voxel size was adjusted to the extended peritumor region by convolving the ROI with the 3D box kernel. The Simple_ITK package in Python software (version 3.6) was then used to obtain the peritumor region by expanding the intratumor ROI by 3 mm and 5 mm in 3D. Notably, regions covered by the expansion that were not the tumor itself were manually excluded. Two radiologists delineated ROIs in 30 randomized cases and underwent interobserver ICC analysis, retaining features with ICC > 0.75. The ROI segmentation is shown in Fig. 3a.

### Radiomics feature extraction and selection

We used features extracted from Pyradiomics (http://pyradiomics.readthedocs.io.), including first-order (*n* = 234), texture analysis features (*n* = 949), and shape (*n* = 14). 1147 features were extracted from each sequence (including SR-DWI, SR-T2WI) for each ROI (including intratumor, peri-3mm, and peri-5mm). Next, the features of each sequence in each region were combined to obtain multi-sequences of INTRA (*n* = 2394), multi-sequences of peri-3mm (*n* = 2394), and multi-sequences of peri-5mm (*n* = 2394). Finally, a total of 7182 features were acquired. Please refer to Supplementary Tables S2 and S3 for details.

First, Mann–Whitney *U* test and feature filtering were performed on all radiological features, retaining only those with *p* < 0.05. Subsequently, optimal features were selected: Pearson’s rank correlation coefficient calculated inter-feature correlation, with one feature excluded if pairwise correlation > 0.9. Next, LASSO with 10-fold cross-validation selected optimal features (non-zero coefficients via optimal penalty parameter), followed by mRMR for further dimensionality reduction.

### Radiomics models and clinical model construction

After 5-fold cross-validation, retained features were used to build DWI_T2WI_INTRA, DWI_T2WI_IntraPeri3mm, and DWI_T2WI_IntraPeri5mm models via logistic regression. ROC curves were plotted, AUC (95% CI) calculated, and DeLong tests done to compare AUCs. Significant clinical features from Table 2 were used to build clinical models by Random Forest. The optimal multiparameter multiregional model was combined with the clinical model to construct a fusion model, with the same evaluation, calibration curves, DCA plotted. The workflow is shown in Fig. 3b.

**Table 2:** Clinical baseline information of all patients

Characteristic	Training cohort (*n* = 135)			Internal validation cohort (*n* = 59)			External test cohort (*n* = 49)		
	LN metastasis (−)	LN metastasis (+)	*p*-value	LN metastasis (−)	LN metastasis (+)	*p*-value	LN metastasis (−)	LN metastasis (+)	*p*-value
Age (years)	62.73 ± 10.55	65.48 ± 9.49	0.14	62.75 ± 8.94	61.87 ± 10.40	0.75	64.92 ± 14.07	58.51 ± 11.38	0.12
Tumor length (cm)	4.23 ± 1.63	5.42 ± 4.58	0.07	4.54 ± 1.99	4.29 ± 1.99	0.46	4.55 ± 1.19	5.42 ± 4.94	0.81
Max diameter (mm)	32.02 ± 5.86	31.22 ± 8.66	1.00	32.60 ± 5.60	30.54 ± 7.98	0.31	33.33 ± 5.30	29.38 ± 8.89	0.16
Gender			0.19			0.25			1.00
Male	31 (77.50)	61 (64.21)		16 (80.00)	24 (61.54)		7 (58.33)	21 (56.76)	
Female	9 (22.50)	34 (35.79)		4 (20.00)	15 (38.46)		5 (41.67)	16 (43.24)	
Underlying disease			0.51			0.46			0.052
Absence	20 (50.00)	55 (57.89)		8 (40.00)	21 (53.85)		4 (33.33)	26 (70.27)	
Existence	20 (50.00)	40 (42.11)		12 (60.00)	18 (46.15)		8 (66.67)	11 (29.73)	
mrT stage			0.38			0.53			0.58
T2	9 (22.50)	13 (13.68)		2 (10.00)	8 (20.51)		1 (8.33)	7 (18.92)	
T3	25 (62.50)	70 (73.68)		16 (80.00)	26 (66.67)		9 (75.00)	22 (59.46)	
T4	6 (15.00)	12 (12.63)		2 (10.00)	5 (12.82)		2 (16.67)	8 (21.62)	
Tumor differentiation			< 0.001*			0.07			0.46
Low	2 (5.00)	15 (15.79)		1 (5.00)	10 (25.64)		1 (8.33)	4 (10.81)	
Middle	27 (67.50)	77 (81.05)		18 (90.00)	29 (74.36)		9 (75.00)	31 (83.78)	
High	11 (27.50)	3 (3.16)		1 (5.00)	0 (0.00)		2 (16.67)	2 (5.41)	
Distant metastasis			1.00			0.91			0.67
Negative	34 (85.00)	81 (85.26)		17 (85.00)	35 (89.74)		8 (66.67)	29 (78.38)	
Positive	6 (15.00)	14 (14.74)		3 (15.00)	4 (10.26)		4 (33.33)	8 (21.62)	
EMVI			0.08			0.46			0.55
Negative	29 (72.50)	52 (54.74)		12 (60.00)	18 (46.15)		4 (33.33)	18 (48.65)	
Positive	11 (27.50)	43 (45.26)		8 (40.00)	21 (53.85)		8 (66.67)	19 (51.35)	
MRF			0.45			0.32			1.00
Negative	25 (62.50)	51 (53.68)		11 (55.00)	28 (71.79)		6 (50.00)	18 (48.65)	
Positive	15 (37.50)	44 (46.32)		9 (45.00)	11 (28.21)		6 (50.00)	19 (51.35)	
Tumor location			0.90			0.25			0.71
Low	13 (32.50)	28 (29.47)		6 (30.00)	18 (46.15)		4 (33.33)	11 (29.73)	
Middle	19 (47.50)	45 (47.37)		9 (45.00)	17 (43.59)		5 (41.67)	20 (54.05)	
High	8 (20.00)	22 (23.16)		5 (25.00)	4 (10.26)		3 (25.00)	6 (16.22)	
CEA			0.58			0.86			1.00
< 5 ng/mL	23 (57.50)	48 (50.53)		9 (45.00)	20 (51.28)		6 (50.00)	17 (45.95)	
> 5 ng/mL	17 (42.50)	47 (49.47)		11 (55.00)	19 (48.72)		6 (50.00)	20 (54.05)	
CA125			0.29			1.00			0.68
< 35 U/mL	35 (87.50)	74 (77.89)		18 (90.00)	35 (89.74)		11 (91.67)	30 (81.08)	
> 35 U/mL	5 (12.50)	21 (22.11)		2 (10.00)	4 (10.26)		1 (8.33)	7 (18.92)	
CA19-9			0.99			1.00			0.11
< 37 U/mL	34 (85.00)	79 (83.16)		17 (85.00)	32 (82.05)		10 (83.33)	19 (51.35)	
> 37 U/mL	6 (15.00)	16 (16.84)		3 (15.00)	7 (17.95)		2 (16.67)	18 (48.65)	
WBC (10^9^/L)			0.29			0.89			0.96
< 4	0 (0.00)	3 (3.16)		1 (5.00)	1 (2.56)		1 (8.33)	3 (8.11)	
4–10	36 (90.00)	76 (80.00)		17 (85.00)	34 (87.18)		9 (75.00)	29 (78.38)	
> 10	4 (10.00)	16 (16.84)		2 (10.00)	4 (10.26)		2 (16.67)	5 (13.51)	
N (10^9^/L)			0.31			0.72			0.70
< 2	0 (0.00)	4 (4.21)		0 (0.00)	1 (2.56)		0 (0.00)	2 (5.41)	
2–7	34 (85.00)	72 (75.79)		18 (90.00)	33 (84.62)		10 (83.33)	30 (81.08)	
> 7	6 (15.00)	19 (20.00)		2 (10.00)	5 (12.82)		2 (16.67)	5 (13.51)	
L (10^9^/L)			0.29			0.25			0.49
< 0.7	3 (7.50)	6 (6.32)		0 (0.00)	4 (10.26)		0 (0.00)	3 (8.11)	
0.7–4	36 (90.00)	89 (93.68)		20 (100.00)	34 (87.18)		12 (100.00)	33 (89.19)	
> 4	1 (2.50)	0 (0.00)		0 (0.00)	1 (2.56)		0 (0.00)	1 (2.70)	
Neu (%)			0.21			0.71			0.028*
< 50	0 (0.00)	7 (7.37)		1 (5.00)	3 (7.69)		2 (16.67)	0 (0.00)	
50–70	27 (67.50)	60 (63.16)		14 (70.00)	23 (58.97)		8 (66.67)	24 (64.86)	
> 70	13 (32.50)	28 (29.47)		5 (25.00)	13 (33.33)		2 (16.67)	13 (35.14)	
Lym (%)			0.85			0.07			0.37
< 20	11 (27.50)	24 (25.26)		3 (15.00)	13 (33.33)		2 (16.67)	11 (29.73)	
20–40	26 (65.00)	61 (64.21)		17 (85.00)	22 (56.41)		8 (66.67)	24 (64.86)	
> 40	3 (7.50)	10 (10.53)		0 (0.00)	4 (10.26)		2 (16.67)	2 (5.41)	
Tumor thrombus			0.028*			0.35			0.55
Negative	35 (87.50)	64 (67.37)		15 (75.00)	23 (58.97)		8 (66.67)	19 (51.35)	
Positive	5 (12.50)	31 (32.63)		5 (25.00)	16 (41.03)		4 (33.33)	18 (48.65)	
LVI			0.023*			0.013*			0.35
Negative	29 (72.50)	47 (49.47)		18 (90.00)	21 (53.85)		9 (75.00)	20 (54.05)	
Positive	11 (27.50)	48 (50.53)		2 (10.00)	18 (46.15)		3 (25.00)	17 (45.95)	
PNI			0.001*			0.042*			0.08
Negative	21 (52.50)	21 (22.11)		10 (50.00)	8 (20.51)		8 (66.67)	12 (32.43)	
Positive	19 (47.50)	74 (77.89)		10 (50.00)	31 (79.49)		4 (33.33)	25 (67.57)	
Smoke			1.00			0.57			1.00
Absence	28 (70.00)	68 (71.58)		16 (80.00)	27 (69.23)		9 (75.00)	29 (78.38)	
Existence	12 (30.00)	27 (28.42)		4 (20.00)	12 (30.77)		3 (25.00)	8 (21.62)	
Alcoholism			0.72			1.00			0.11
Absence	28 (70.00)	71 (74.74)		16 (80.00)	30 (76.92)		12 (100.00)	27 (72.97)	
Existence	12 (30.00)	24 (25.26)		4 (20.00)	9 (23.08)		0 (0.00)	10 (27.03)	

Underlying disease: suffering from hypertension and diabetes

*PNI* perineural invasion, *LVI* lymphatic vessel invasion, *CEA* carcinoembryonic antigen, *WBC* white blood cell count, *N* NEUT#, *L* LYMPH#, *Neu* NEUT%, *Lym* LYMPH%

* *p* < 0.05

Data analysis was performed using Python 3.7.12 on the OneKey platform version 3.5.12. and machine learning algorithms (LR), including LASSO, were implemented via Scikit-learn version 1.0.2.

### Statistical analysis

Continuous variables are presented as mean ± SD (*t*-test/Mann–Whitney *U* test), while categorical variables are shown as frequencies (%) ($\chi$²test). Ordinal data are expressed as median (IQR). The Wilcoxon signed-rank test was used for qualitative comparisons. Inter-reader agreement (kappa statistics) was categorized as: slight (0–0.20), fair (0.21–0.40), moderate (0.41–0.60), substantial (0.61–0.80), or near-perfect (0.81–1.0). Statistical significance was set at *p* < 0.05. All statistical analyses were completed on the OneKey platform version 3.5.12 using version 0.13.2 statistical modeling, except for the Wilcoxon signed-rank test and kappa statistical analysis using SPSS (Version 26.0.).

## Results

### Patients characteristics

A total of 243 patients were included in this study, 66% male and 34% female, mean age 63.14 ± 10.50. All patients were divided into a training cohort of 135 (95 LNM+, 40 LNM−), an internal validation cohort of 59 (39 LNM+, 20 LNM−) and an external test cohort of 49 (37 LNM+, 12 LNM−). In both the training cohort and the internal validation cohort, there were statistically significant differences in tumor differentiation, lymphatic vessel invasion (LVI), and perineural invasion (PNI) between the LNM (−) and the LNM (+), whereas NEUT% had a statistically significant difference in the external test cohort, with no statistical significance in other clinical parameters. Detailed patient characteristics are summarized in Table 2.

### Image qualitative analysis

As shown in Table 3, the Wilcoxon signed-rank test confirmed that both SR-DWI and SR-T2WI were significantly better than the original images (ORI) in all five quality metrics, including anatomical visualization, lesion clarity, organ contrast, mosaic effect, and overall quality (all *p* < 0.001). Notably, although the median lesion conspicuity scores of SR-DWI and ORI-DWI were the same [3 (IQR 1)], their distributions differed significantly (*p* < 0.001).

**Table 3:** Qualitative visual scoring scale

Characteristic	SR-T2WI	ORI-T2WI	*p*-value	SR-DWI	ORI-DWI	*p*-value
Anatomical visualization of the rectum	4 (1)	3 (1)	< 0.001*	3 (1)	2 (1)	< 0.001*
Rectal lesion conspicuity	4 (1)	3 (1)	< 0.001*	3 (1)	3 (1)	< 0.001*
Contrast with neighboring organs	4 (1)	2 (1)	< 0.001*	3 (0)	2 (1)	< 0.001*
Mosaic effect	4 (1)	2 (1)	< 0.001*	4 (1)	2 (1)	< 0.001*
Overall image quality	4 (1)	3 (1)	< 0.001*	3 (1)	2 (1)	< 0.001*

Data are medians with interquartile ranges in parentheses. “SR” means “super-resolution,” and “ORI” means “original”

* *p* < 0.05

**Fig. 4:**
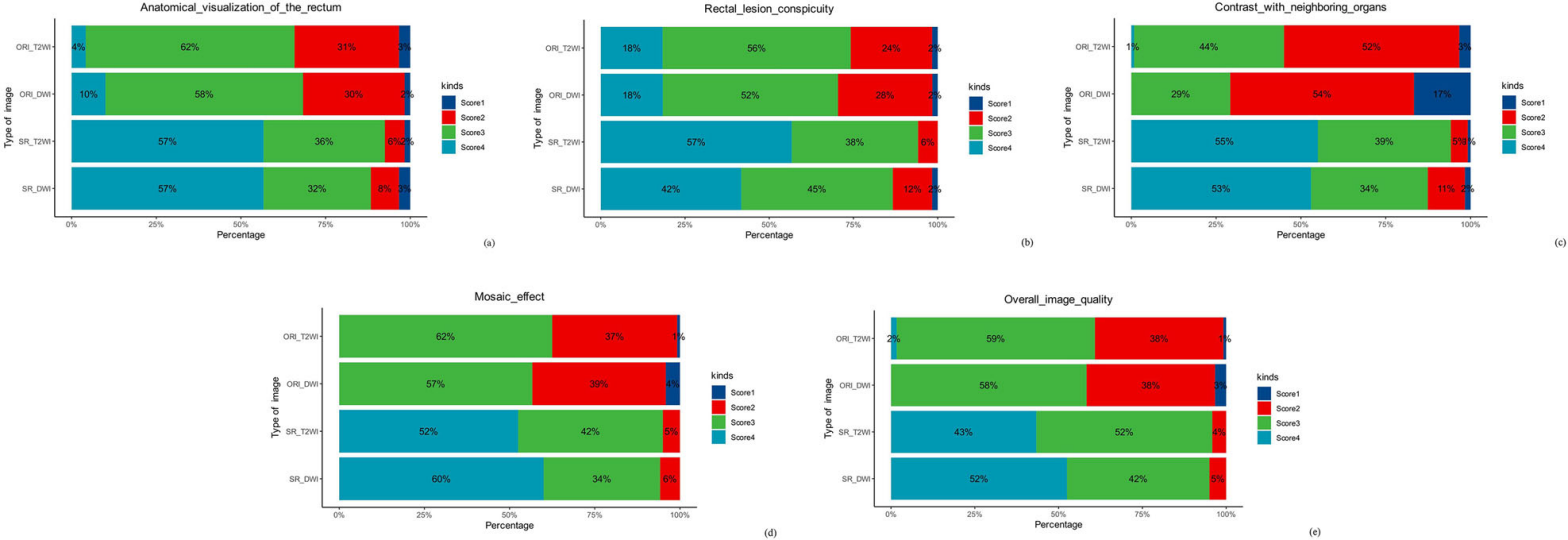
Percentage distributions of scores for each indicator. **a** Anatomical visualization of the rectum, **b** rectal lesion conspicuity, **c** contrast with neighboring organs, **d** mosaic effect, **e** overall quality. Color code: dark blue = 1 point; red = 2 points; green = 3 points; light blue = 4 points

Inter-reader agreement analysis revealed substantial consistency between two radiologists in their quantitative assessments of DWI and T2WI sequences. For DWI (Cohen’s κ = 0.62–0.71, all *p* < 0.001), anatomical visualization of the rectum of 0.71, rectal lesion conspicuity of 0.62, contrast with neighboring organs of 0.70, mosaic effect of 0.69, and overall image quality of 0.65. For T2WI (Cohen’s κ = 0.61–0.71, all *p* < 0.001), anatomical visualization of the rectum of 0.68, rectal lesion conspicuity of 0.61, contrast with neighboring organs of 0.65, mosaic effect of 0.71, overall image quality of 0.68. The distribution of scores is visualized in Fig. 4.

### Clinical risk features and clinical model construction

The retained clinically independent risk factors associated with LNM after univariate and multivariate analyses were tumor differentiation, PNI, tumor thrombus, and LVI, as shown in Table 4. The clinical model in each cohort was 0.824 (95% CI: 0.748–0.902), 0.697 (95% CI: 0.558–0.837) and 0.660 (95% CI: 0.492–0.828), respectively.

**Table 4:** The univariate and multivariate logistic regression analysis of risk factors for LNM in all patients

Univariate analysis				Multivariate analysis			
Characteristic	OR	95% CI	*p*-value	Characteristic	OR	95% CI	*p*-value
Age	1.014	1.009–1.019	0				
Max diameter	1.026	1.016–1.036	0				
Tumor length	1.212	1.133–1.297	0				
mrT stage	1.337	1.206–1.483	0				
Tumor differentiation	1.395	1.202–1.619	0	Tumor differentiation	0.121	0.041–0.357	0.001*
Neu	1.415	1.237–1.618	0				
N	1.477	1.280–1.704	0				
WBC	1.498	1.296–1.733	0				
Tumor location	1.518	1.298–1.775	0				
L	1.545	1.318–1.809	0				
Lym	1.568	1.328–1.850	0				
MRF	2.933	1.793–4.797	0				
Underlying disease	2	1.275–3.139	0.011				
Ca19-9	2.667	1.213–5.859	0.04				
Distant metastasis	2.333	1.046–5.207	0.082				
Smoke	2.25	1.271–3.983	0.019				
Alcoholism	2	1.119–3.579	0.05				
CEA	2.765	1.735–4.402	0				
CA125	4.2	1.853–9.526	0.004				
PNI	3.895	2.552–5.948	0	PNI	6.597	2.474–17.584	0.002*
EMVI	3.909	2.243–6.814	0				
Tumor thrombus	6.2	2.807–13.695	0	Tumor thrombus	5.737	1.513–21.758	0.031*
Gender	3.778	2.0040–7.001	0				
LVI	4.364	2.517–7.561	0	LVI	4.688	1.687–13.027	0.013*

* *p* < 0.05

### Multisequence, multiregion radiomics models and combined model construction and comparison

Tumor segmentation had good intra-observer reproducibility. Following dimension reduction via mRMR and LASSO, 12, 15 and 16 highly robust features were retrained (e.g., Fig. S2) for training DWI_T2WI_INTRA, DWI_T2WI_IntraPeri3mm and DWI_T2WI_IntraPeri5mm models, respectively.

The AUC of the three independent LNM prediction models we built ranged from 0.572 to 0.880 in cohorts. First, the DWI_T2WI_INTRA model performed in every cohort was 0.842 (95% CI: 0.774–0.910), 0.644 (95% CI: 0.494–0.794), and 0.572 (95% CI: 0.359–0.786), respectively. In the two DWI_T2WI fusion models, the DWI_T2WI_IntraPeri3mm model achieved 0.880 (95% CI: 0.821–0.938), 0.735 (95% CI: 0.604–0.865), and 0.714 (95% CI: 0.530–0.898), respectively. The AUC of the DWI_T2WI_IntraPeri5mm model in each cohort then exhibited 0.865, 0.705, and 0.653. The ROC curves, results of the diagnostic ability and the DeLong test for each model are demonstrated in Fig. S3 and Table S4.

**Fig. 5:**
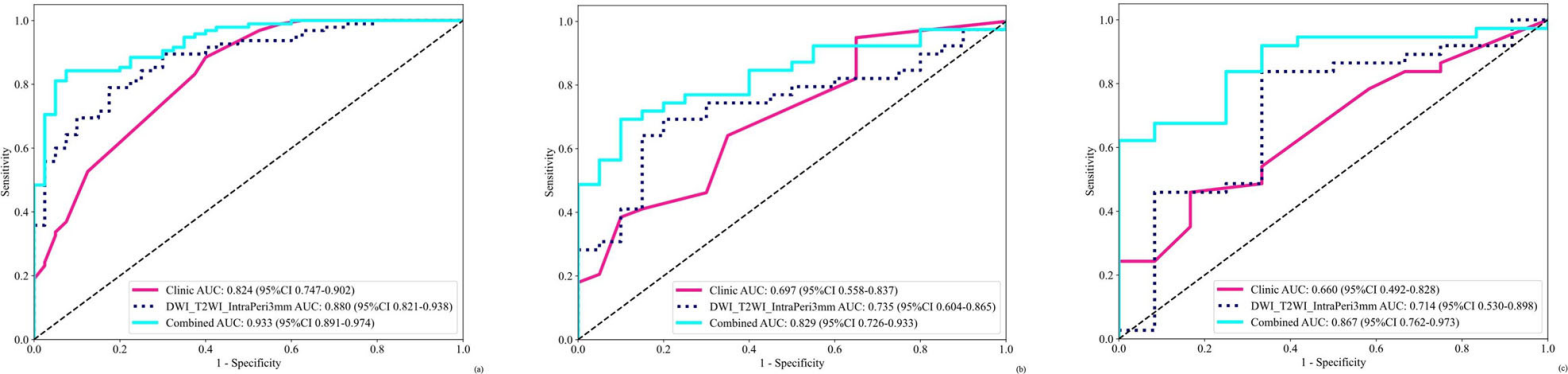
ROC curves for training cohort (**a**), internal validation cohort (**b**), and external test cohort (**c**) of three models. “Combined” denotes Clinical + InterPeri3mm. Line styles: pink solid = Clinic; dark blue dashed = DWI_T2WI_IntraPeri3mm; light blue solid = Combined

**Fig. 6:**
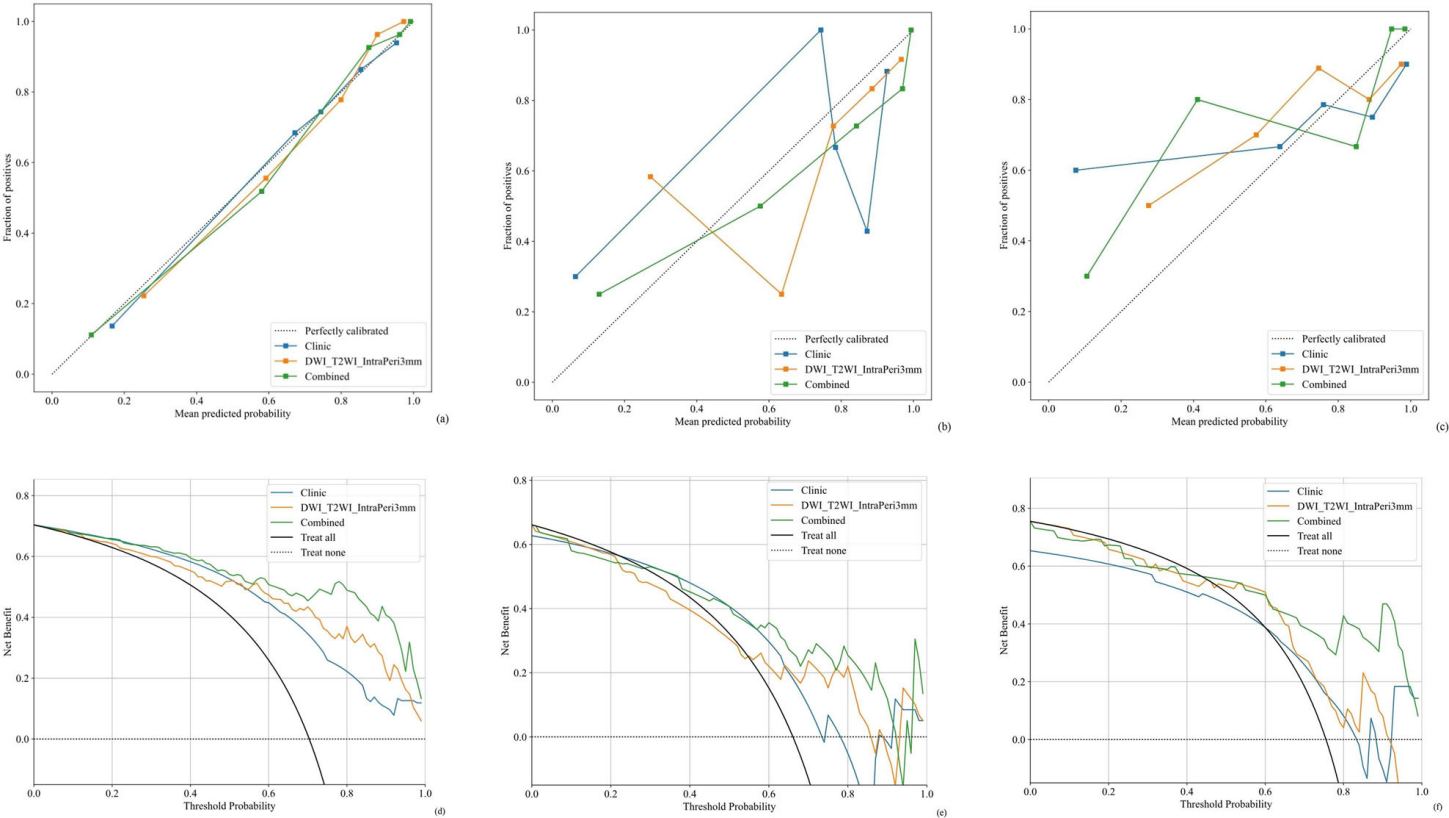
Calibration curves for training cohort (**a**), internal validation cohort (**b**), and external test cohort (**c**) of three models. Color code: blue = clinical model; orange = DWI_T2WI_IntraPeri3mm; green = combined model. Closer alignment of green lines with dashed diagonals in training/internal validation cohorts demonstrates accurate predictions, while poorer performance occurs in the test cohort; DCA for training cohort (**d**), internal validation cohort (**e**), and external test cohort (**f**) of three models. Axes: *x* = threshold probability; *y* = net benefit. Color code: blue = clinical model; orange = DWI_T2WI_IntraPeri3mm; green = combined model. Notably, the combined model achieves higher net benefits than the other two models across major threshold probabilities, particularly in training and internal validation cohorts

The combined models (DWI_T2WI_IntraPeri3mm + clinical risk factors) achieved 0.933 (95% CI: 0.891–0.974), 0.829 (95% CI: 0.604–0.865) and 0.867 (95% CI. 0.762–0.973) in the training, internal validation, and external test cohorts respectively, outperforming standalone DWI_T2WI_IntraPeri3mm model and the clinical model across all cohorts (0.933 vs. 0.880 vs. 0.824, 0.829 vs. 0.735 vs. 0.697, and 0.867 vs. 0.714 vs. 0.660, respectively). The ROC curve, Calibration curve and DCA curve for each model are shown in Figs. 5 and 6, and other details as described in Table 5.

**Table 5:** The diagnostic performance of the clinic model, the DWI_T2WI_IntraPeri3mm model, and the combined model on the train, internal validation and external test cohort for predicting LNM

Model	AUC	95% CI	*p*1	*p*2	Accuracy	Sensitivity	Specificity	PPV	NPV	Cohort
Clinic	0.824	0.748–0.902			0.880	0.884	0.600	0.840	0.686	Train
DWI_T2WI_IntraPeri3mm	0.880	0.821–0.939	0.28		0. 880	0.789	0.825	0.915	0.623	Train
Combined	0.933	0.891–0.974	0.005*	0.053	0.867	0.842	0.925	0.964	0.712	Train
Clinic	0.697	0.558–0.837			0.746	0.949	0.350	0.740	0.778	Internal validation
DWI_T2WI_IntraPeri3mm	0.735	0.604–0.865	0.67		0.729	0.692	0.800	0.871	0.571	Internal validation
Combined	0.829	0.727–0.933	0.052	0.037*	0.763	0.692	0.900	0.931	0.600	Internal validation
Clinic	0.660	0.492–0.828			0.551	0.459	0.833	0.895	0.333	External test
DWI_T2WI_IntraPeri3mm	0.714	0.530–0.898	0.73		0.796	0.838	0.667	0.886	0.571	External test
Combined	0.867	0.762–0.973	0.035*	0.027*	0.714	0.622	1.000	1.000	0.462	External test

*p*1: Clinic model compared with other models; *p*2: DWI_T2WI_IntraPeri3mm compared with other models, *p* < 0.05 indicates statistical significance

* *p* < 0.05

## Discussion

Based on SR-MR images meeting clinical diagnostic requirements, which were obtained via GAN-based 3D medical image reconstruction, this study developed and validated the performance of multisequence, multiregion radiomics models using DWI and T2WI, as well as a combined MRI radiomics-clinical model, for predicting preoperative LNM in RC. The results demonstrated that multiregion models outperformed intratumoral-only models under multiparameter fusion. This study suggests that multiparameter, multiregional MRI radiomics—a relatively noninvasive assessment method—along with MRI radiomic-clinical modeling, can further enhance diagnostic efficacy for LNM.

In clinical practice, colorectal cancer treatment decision-making relies heavily on imaging assessment. Differentiation of treatment strategies determines patient prognosis [22]. For example, according to the recommendations of the 2020 NCCN Guidelines, the implementation of preoperative nCRT is primarily based on the presence of LNM [23]. The differences between preoperative MRI-based N-staging and postoperative pathology results highlight the limitations of positive LNs imaging features in reliably assessing LNM and may lead to excessive staging, overtreatment, and increased complications [24]. Consequently, our retrospective study used postoperatively pathologically confirmed mesorectal LNM(+) to ensure model predictive power. We developed predictive models integrating tumor and peritumoral imaging features, as well as tumor-peritumoral regional imaging histology, for preoperative risk assessment of mesorectal LNM in RC patients, aiming to reduce overtreatment and improve survival quality.

In order to improve the performance of the prediction model, we focused on the fact that image quality can be improved using GAN-based super-resolution algorithms—a novel architecture that fuses the channel attention mechanisms with residual dense blocks [25], replacing the traditional relative average discriminator and improving the loss function of the generator to enhance the peak signal-to-noise ratio (PSIM) and structural similarity index (SSIM) for lesion characterization. Conditional GAN can be applied to reconstruct two-dimensional T2WI into isotropic volumetric data [26], demonstrating measurable improvements in multiple quantitative parameters, including SSIM and RMSE. Therefore, we have attempted this GAN-based 3D medical image reconstruction technique to improve the quality of MR images. Recent studies based on this technique have compared the use of standard image prediction models with super-resolution (SR) image prediction models and have shown that SR-based models perform better [27, 28]. Our qualitative assessment showed that SR images outperformed the original images with good inter-reader agreement. These findings support that SR images can be used as a reliable tool for clinical image diagnosis and the development of predictive models for LNM of RC.

MRI histology has been shown to significantly outperform CT-based radiomics models in predicting LNM in RC [29]. In conventional MRI studies, Zhou et al [30] integrated the tumor histogram features from conventional DWI/T2WI and clinical risk factors to construct a combined model (AUC = 0.860), which outperformed their single-sequence model, In contrast, our combined model—built by integrating features, clinical risk factors, and data from SR-DWI/SR-T2WI—exhibited significantly improved performance (AUC = 0.933). As the depth and breadth of the various research continue to expand, some investigators have found the heterogeneity of peritumor tissue biology and the complexity of its microenvironment, taking the peritumor region into consideration. Li et al [31] incorporated the features of the peri_3mm, and the results verified that the intra and peritumoral model efficacy (AUC = 0.760) was significantly higher when compared to the intratumor (AUC = 0.640) or peritumor (AUC = 0.680) models alone; this study did not consider combining DWI. In contrast, Meng et al [32] chose to apply tumor-rectal mesenteric fascial fat region and combined multiple sequences (AUC = 0.87). Both used conventional images. Our study is based on the combination of multiple sequences and multiple regions of SR, especially the IntraPeri3mm model (AUC = 0.880), which slightly outperformed the former two, and the predictive performance of the model was improved with the combined clinical model (AUC = 0.933).

The presence of tumor cell metastasis and micro-metastasis in regional LNs is regarded as a key reason for local recurrence and distant metastasis following surgical treatment of RC [33, 34], and rectal mesenteric LNs are the most frequent sites of metastasis in RC [35]. Therefore, constructing a prediction model by combining peritumor multiregional features is beneficial for clinical decision-making to predict the LN status. We not only chose the peri-3mm region as one of the elements for constructing the model, but also expanded it to 5mm to construct model as well. Compared with the DWI_T2WI_ INTRA model, the DWI_T2WI_ IntraPeri5mm model had a higher AUC than in the training cohort, internal validation cohort, and external test cohort (AUC: 0.865 vs. 0.842, *p* = 0.44; 0.705 vs. 0.644, *p* = 0.31; 0.653 vs. 0.572, *p* = 0.28). Although the DeLong test was not statistically significant, in the internal validation cohort, the DWI_T2WI_IntraPeri5mm model had higher sensitivity, specificity, and accuracy than the DWI_T2WI_INTRA model. The DWI_T2WI_IntraPeri3mm model performed well in AUC, sensitivity, and accuracy, except that it was inferior to the DWI_T2WI_IntraPeri5mm model in specificity in the external test cohort (0.667 vs. 0.917).

Further, we incorporated clinically independent risk factors (tumor differentiation, tumor thrombus, LVI, PNI) based on clinical characteristics. Our combined model—integrating the DWI_T2WI_IntraPeri3mm model and these risk factors—exhibited increased AUC without statistical significance in the training cohort; however, significant differences were observed in the internal validation and external test cohorts. This suggests the combined model enhances LNM prediction in RC patients beyond radiomics alone and holds clinical utility potential. In addition, previous studies [30, 36] have reported that serologic indicators such as CEA, CA19-9, and WBC can be used as risk factors for the prediction of LNM status in RC, however, despite collecting multiple serological markers, only NEUT% showed significant statistical difference (*p* = 0.028) in the external test cohort, which does not mean that serologic markers have no predictive power, which may be related to demographic differences in the study populations we included or to the sample size. Accordingly, the predictive performance of clinical models for LNM cannot be generalized and should be interpreted with caution. The inclusion of LVI and PNI supports the notion that peritumoral radiological features can enhance predictive performance.

This study has several limitations. First, although multicenter, the small external test cohort limits predictive model generalizability. Variations in MRI parameters (b-value, TR, TE) and demographic differences (lifestyle, socioeconomic factors) between centers may further restrict model generalizability. Potential patient selection bias persists. Thus, broader population validation is needed to ensure clinical utility. Second, the 10-fold cross-validation (CV) used in this study has certain limitations compared to nested CV. Its performance metrics may be prone to over-optimism and inflation. This bias arises because the same data may be used for hyperparameter tuning, feature selection, and final evaluation, potentially leading to overestimation of the model’s true generalizability. Thus, it is necessary to employ a nested CV in future research to ensure clinical applicability. Third, the SR technique improves anatomical clarity but increases the complexity of segmentation and feature extraction. Continuous advances, such as those reported by Zhang et al [37], imply improvements in this step. Finally, the exclusion of LN(+)-specific radiomic features may constrain predictive model performance. Research indicates that current radiomics approaches show a lack of convincing correspondence between MRI-detected suspicious LNs and postoperative pathological LNs(+), whether the combination of tumor and nodal morphology [38] or only LNs radiomic features [39], resulting in discrepancies between radiology and pathology. In subsequent research, accurate image-histology colocalization enhances diagnostic performance [40], which requires precise collaboration of imaging, surgical localization and pathology to potentially address current technical and analytical limitations, while advancing clinically relevant radiomics applications.

## Conclusion

GAN-based 3D super-resolution reconstruction model for multisequence and multiregion preoperative mesorectal LNM prediction in rectal cancer, especially the DWI_T2WI_IntraPeri3mm model, demonstrated superior diagnostic accuracy. Combining radiomics with clinical data significantly improved the diagnostic performance of the model and shows promise for supporting clinical decision-making.

## Supplementary information

ELECTRONIC SUPPLEMENTARY MATERIAL

## Data Availability

The datasets generated and analyzed during the current study are not publicly available due to the PACS system regulations by the Second Affiliated Hospital of Harbin Medical University and the Fifth Affiliated Hospital of Harbin Medical University, but are available from the corresponding author upon reasonable request.

## Abbreviations

CEA: Carcinoembryonic antigen
CI: Confidence interval
CV: Cross-validation
DWI: Diffusion-weighted imaging
EMVI: Extramural vascular invasion
GANs: Generative adversarial networks
INTRA: Intratumor area
IntraPeri: Intratumor area combined with peritumoral area
LASSO: Least absolute shrinkage and selection operator
LNM: Lymph node metastasis
LNs: Lymph nodes
LVI: Lymphatic vessel invasion
MRF: Mesorectal fascia
mRMR: Maximum relevance minimum redundancy
mrT stage: MRI-based T staging
NEUT%: Neutrophil percentage
ORI: Original
Peri: Peritumoral area
PNI: Perineural invasion
Random Forest: Random Forest algorithm
RC: Rectal cancer
RRRC: Radical resection of rectal cancer
SR: Super resolution
WBC: White blood cell count
