# Supplementary material for: Generative adversarial networks: multiparametric, multiregion super-resolution MRI in predicting lymph node metastasis in rectal cancer
Source: Insights Imaging. 2026 Jan 5;17:1. doi: 10.1186/s13244-025-02173-5 (PMC12770130; doi:10.1186/s13244-025-02173-5)
Supplement: Supplementary file 1 — ELECTRONIC SUPPLEMENTARY MATERIAL [file 13244_2025_2173_MOESM1_ESM.pdf]

Generative Adversarial Networks: Multiparametric, Multi-Region Super-Resolution MRI in Predicting Lymph Node Metastasis in Rectal Cancer

ELECTRONIC SUPPLEMENTARY MATERIAL

Insights Imaging (2025) Wu Y, Jiang T, Liu H, et al.

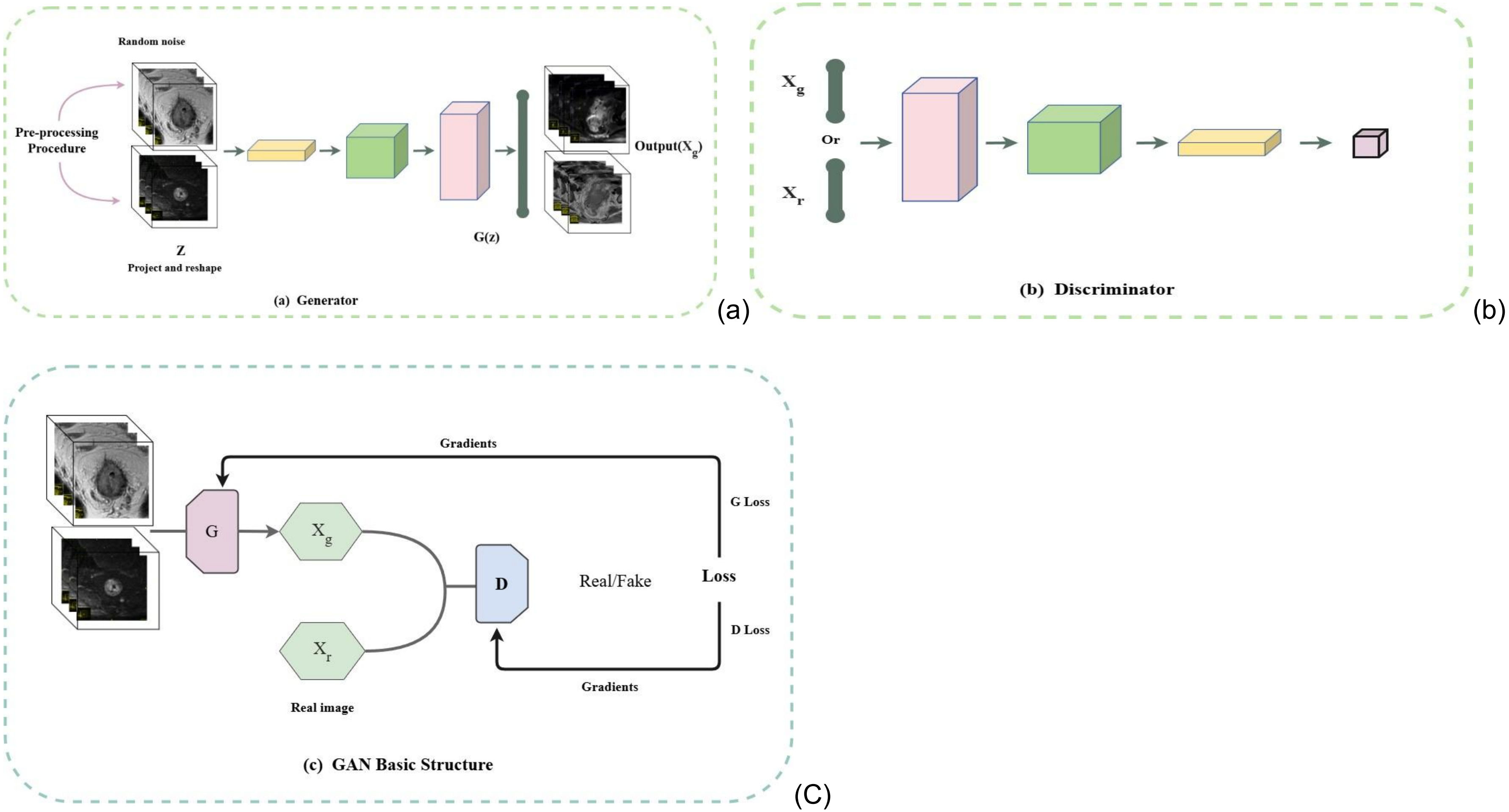

Fig. S1 The basic structure of Generative Adversarial Network (GAN)

Text 1: Details of Radical Resection of Rectal Cancer

Radical Resection of Rectal Cancer—a general term encompassing various radical surgical approaches including Dixon (low anterior resection of rectal cancer), Miles(abdominoperineal resection of rectal cancer, and Hartmann(Hartmann's operation for rectal cancer). The core principle is TME: complete surgical resection of the rectal tumor, invaded surrounding tissues, and regional LNs (typically dissecting up to station 3 LNs).

Text 2: Details of Super-Resolution reconstruction process

The GAN-based 3D super-resolution reconstruction was trained on millions of preprocessed medical images, where noise and artifacts were removed and intensity values normalized. The images are then divided into low-resolution and high-resolution pairs that applied to train the GAN model, where low-resolution images were created through down sampling of the original high-resolution data.

The loss function used in the GAN model consists of three components: gradient loss, L1 loss, and perceptual loss. The gradient loss encourages the generated images to have similar gradient values as the high-resolution images. The L1 loss measures the pixel-wise difference between the generated and high-resolution images. The perceptual loss measures the difference between the feature representations of the generated and high-resolution images obtained from a pre-trained deep learning model. The combination of these loss functions helps to ensure that the generated images are visually similar to the high-resolution images.

Table.S1 Relevant MRI parameters of Center A and Center B

Parameters	T2WI (A)	DWI (A)	T2WI (B)	DWI (B）
Repetition Time (TR)	5990 ms	4881 ms	4200-5500 ms	3000-5000 ms
Echo Time (TE)	125.7 ms	70.2 ms	80-120 ms	50-80 ms
Field of view (FOV)	20 cm	28 cm	24 cm	30 cm
Thickness	3.6 mm	3.6 mm	3.0 mm	3.0 mm
Slice Spacing	0.3 mm	0.3 mm	0 mm	0 mm

Table.S2 List of extracted radiomics features category form areas of INTRA, Peri3mm and Peri5mm

Category	Feature	Meaning
Geometry features (n=14)	Elongation	This group of features include descriptors of the three-dimensional size and shape of the ROI. These features are independent from the gray level intensity distribution in the ROI and are therefore only calculated on the non-derived image and mask.
	Flatness	
	LeastAxisLength	
	MajorAxisLength	
	Maximum2DDiameterColumn	
	Maximum2DDiameterRow	
	Maximum2DDiameterSlice	
	Maximum3DDiameter	
	MeshVolume	
	MinorAxisLength	
	Sphericity	
	SurfaceArea	
	SurfaceVolumeRatio	
	VoxelVolume	
Intensity Features (n=18)	10Percentile	This group of features describe the distribution of voxel intensities within the image region defined by the mask through commonly used and basic metrics.
	90Percentile	
	Energy	
	Entropy	
	InterquartileRange	
	Kurtosis	
	Maximum	
	Mean	
	MeanAbsoluteDeviation	
	Median	
	Minimum	
	Range	
	RobustMeanAbsoluteDeviation	
	RootMeanSquared	
	Skewness	
	TotalEnergy	
	Uniformity	
	Variance	
Texture Features (n=68)	Gray Level Co-occurrence Matrix (GLCM) (n=22)	These features describe the second-order joint probability function of an image region constrained by the mask
	Gray Level Dependence Matrix (GLDM) (n=14)	These features quantify gray level dependencies in an image. A gray level dependency is defined as a the number of connected voxels within distance δ that are dependent on the center voxel.
	Gray Level Run Length Matrix (GLRLM) (n=16)	These features quantify gray level runs, which are defined as the length in number of pixels, of consecutive pixels that have the same gray level value.

Table.S3 List of all 19 filters applied for SR images of INTRA, Peri3mm and Peri5mm.

Image filters (n=19)	Original		original images
	Gradient		Returns the magnitude of the local gradient
	LoG	log_sigma_1_0_mm_3D	The Laplacian of Gaussian filter is an edge enhancement filter.
		log_sigma_2_0_mm_3D	
		log_sigma_3_0_mm_3D	
		log_sigma_4_0_mm_3D	
		log_sigma_5_0_mm_3D	
	Logarithm		Take the logarithm of the image absolute intensity + 1, and scale the value to the original range.
	Exponential		Using e^(absolute intensity) to obtain the index value of intensity, the value is scaled to the original range.
	Square		Take the square of the original pixels and scale them back linearly to the original range.
	Squareroot		Take the square root of the absolute image intensity and scale them back to the original range.
	Wavelet	wavelet_HHH	All combinations of high-pass and low-pass filters in each of the three dimensions (LLH, LHL, LHH, HLL, HLH, HHL, HHH, LLL) are generated
		wavelet_HHL	
		wavelet_HLH	
		wavelet_HLL	
		wavelet_LHH	
		wavelet_LHL	
		wavelet_LLH	
		wavelet_LLL	

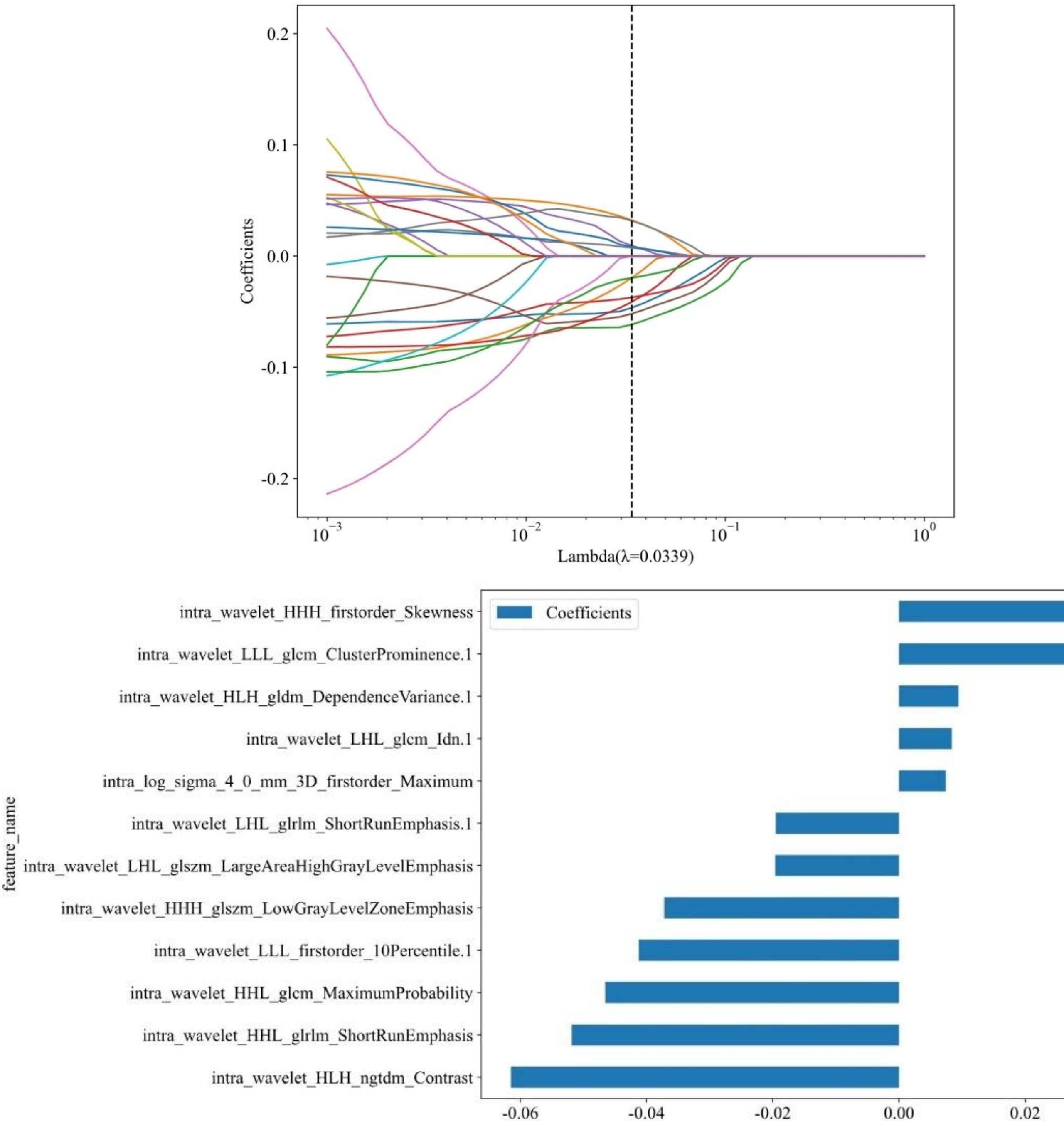
Coefficients
Lambda(λ=0.0339)
feature_name
Coefficients
intra_wavelet_HHH_firstorder_Skewness
intra_wavelet_LLL_glcm_ClusterProminence.1
intra_wavelet_HLH_gldm_DependenceVariance.1
intra_wavelet_LHL_glcm_Idn.1
intra_log_sigma_4_0_mm_3D_firstorder_Maximum
intra_wavelet_LHL_glrlm_ShortRunEmphasis.1
intra_wavelet_LHL_glszm_LargeAreaHighGrayLevelEmphasis
intra_wavelet_HHH_glszm_LowGrayLevelZoneEmphasis
intra_wavelet_LLL_firstorder_10Percentile.1
intra_wavelet_HHL_glcm_MaximumProbability
intra_wavelet_HHL_glrlm_ShortRunEmphasis
intra_wavelet_HLH_ngtdm_Contrast

(a)

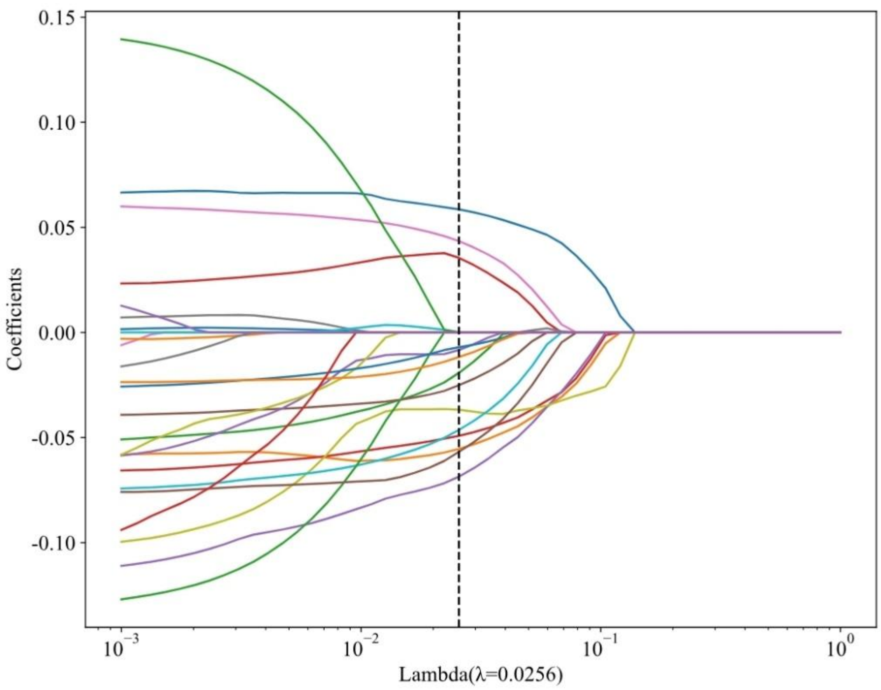

0.15
0.10
0.05
0.00
-0.05
-0.10
Coefficients
10^{-3}
10^{-2}
10^{-1}
10^{0}
Lambda(λ=0.0256)

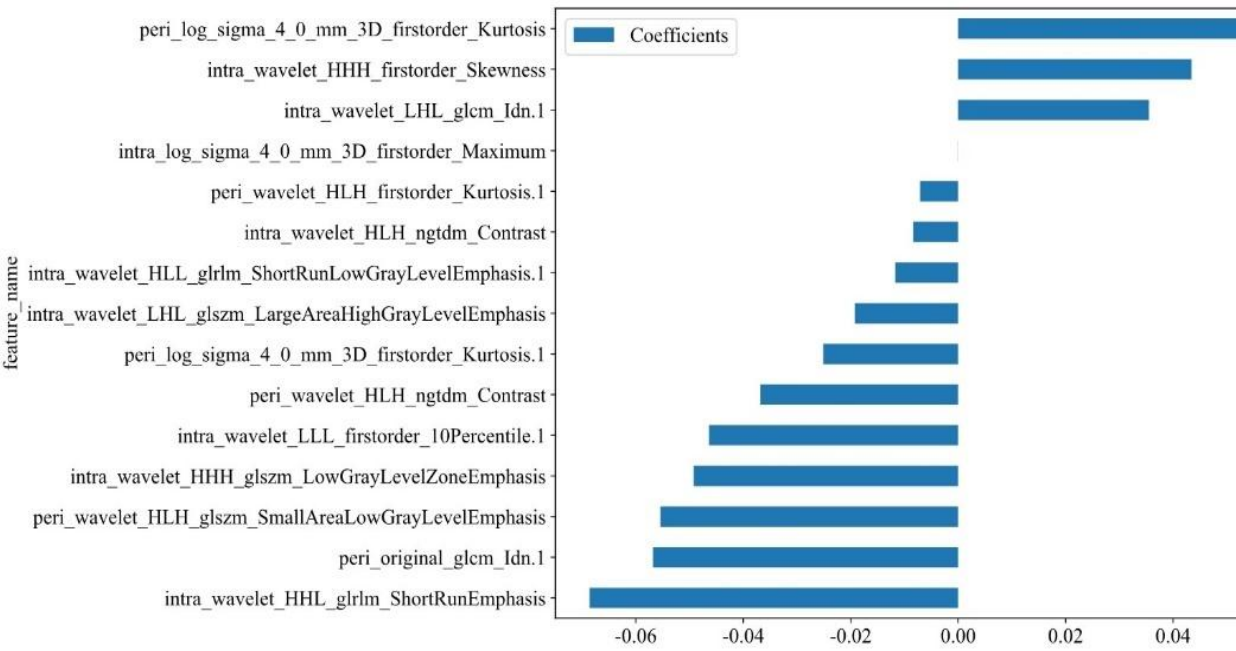

Coefficients
feature_name
peri_log_sigma_4_0_mm_3D_firstorder_Kurtosis
intra_wavelet_HHH_firstorder_Skewness
intra_wavelet_LHL_glcm_Idn.1
intra_log_sigma_4_0_mm_3D_firstorder_Maximum
peri_wavelet_HLH_firstorder_Kurtosis.1
intra_wavelet_HLH_ngtdm_Contrast
intra_wavelet_HLL_glrlm_ShortRunLowGrayLevelEmphasis.1
intra_wavelet_LHL_glszm_LargeAreaHighGrayLevelEmphasis
peri_log_sigma_4_0_mm_3D_firstorder_Kurtosis.1
peri_wavelet_HLH_ngtdm_Contrast
intra_wavelet_LLL_firstorder_10Percentile.1
intra_wavelet_HHH_glszm_LowGrayLevelZoneEmphasis
peri_wavelet_HLH_glszm_SmallAreaLowGrayLevelEmphasis
peri_original_glcm_Idn.1
intra_wavelet_HHL_glrlm_ShortRunEmphasis
-0.06
-0.04
-0.02
0.00
0.02
0.04

(b)

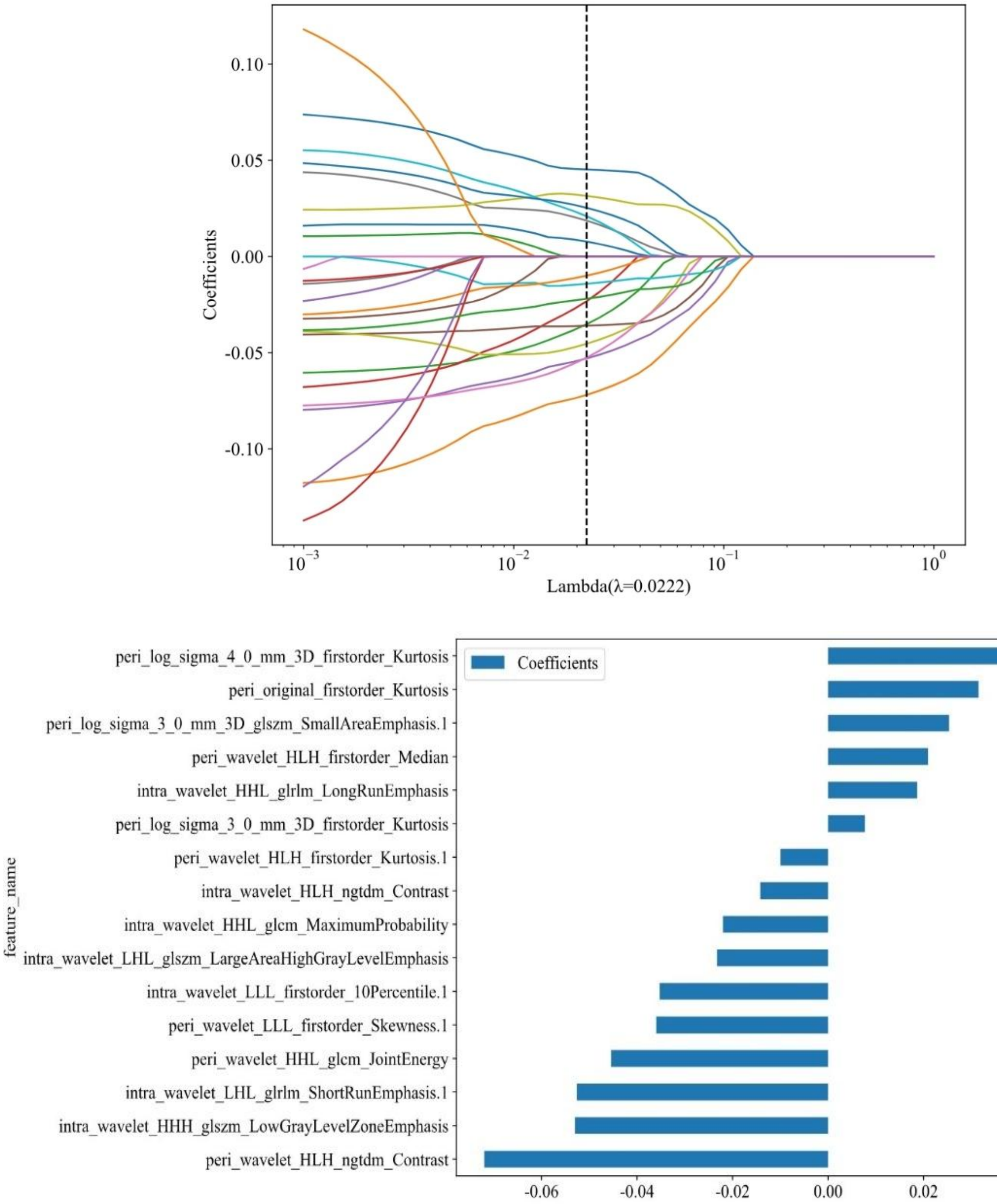

(c)

Fig.S2 Features retained following the mRMR and LASSO approaches include (a) 12 features from DWI_T2WI_INTRA (b) 15 features from DWI_T2WI_IntraPeri3mm (c) 16 features from DWI_T2WI_IntraPeri5mm

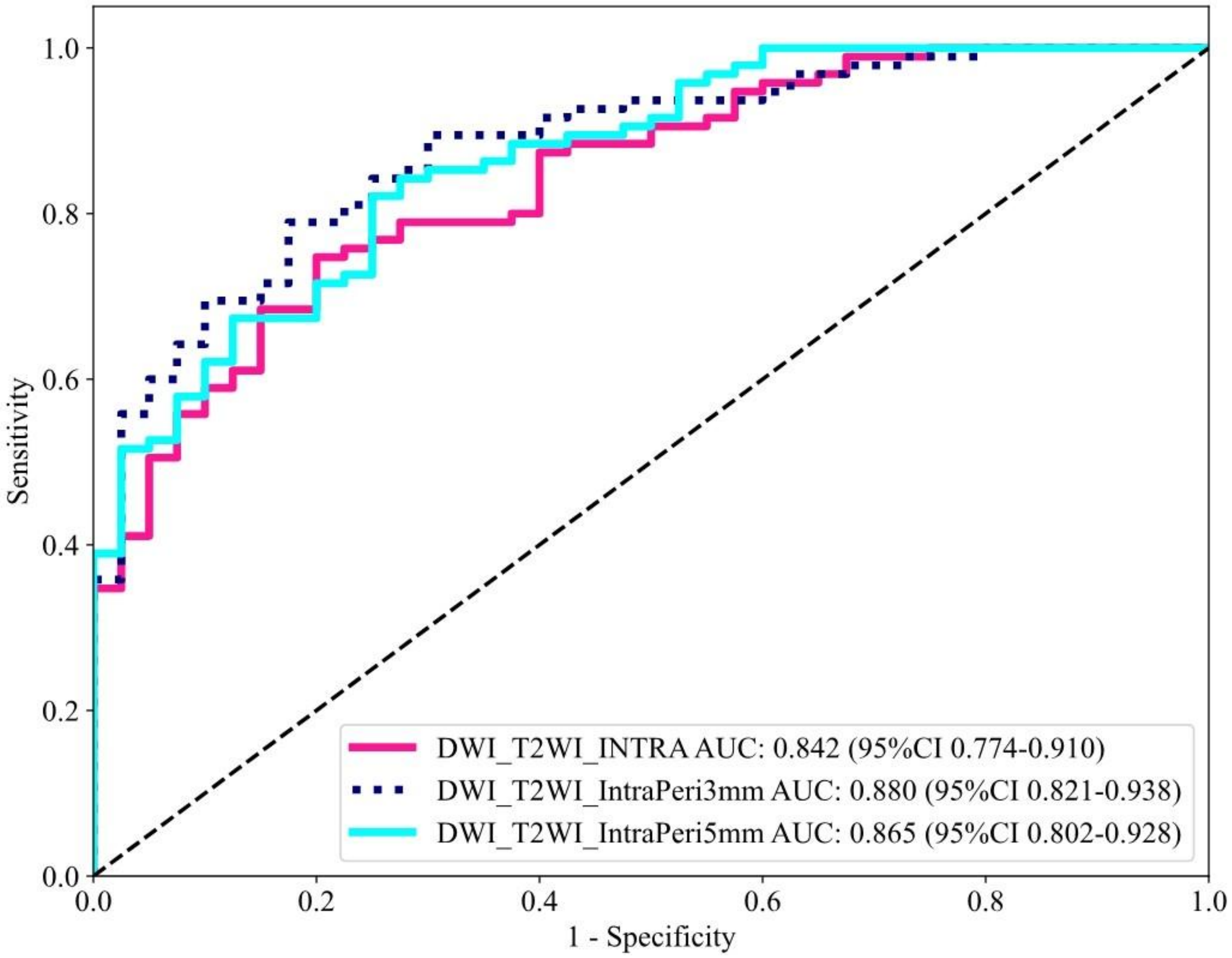

Sensitivity
1 - Specificity
0.0
0.2
0.4
0.6
0.8
1.0
DWI_T2WI_INTRA AUC: 0.842 (95%CI 0.774-0.910)
DWI_T2WI_IntraPeri3mm AUC: 0.880 (95%CI 0.821-0.938)
DWI_T2WI_IntraPeri5mm AUC: 0.865 (95%CI 0.802-0.928)

(a)

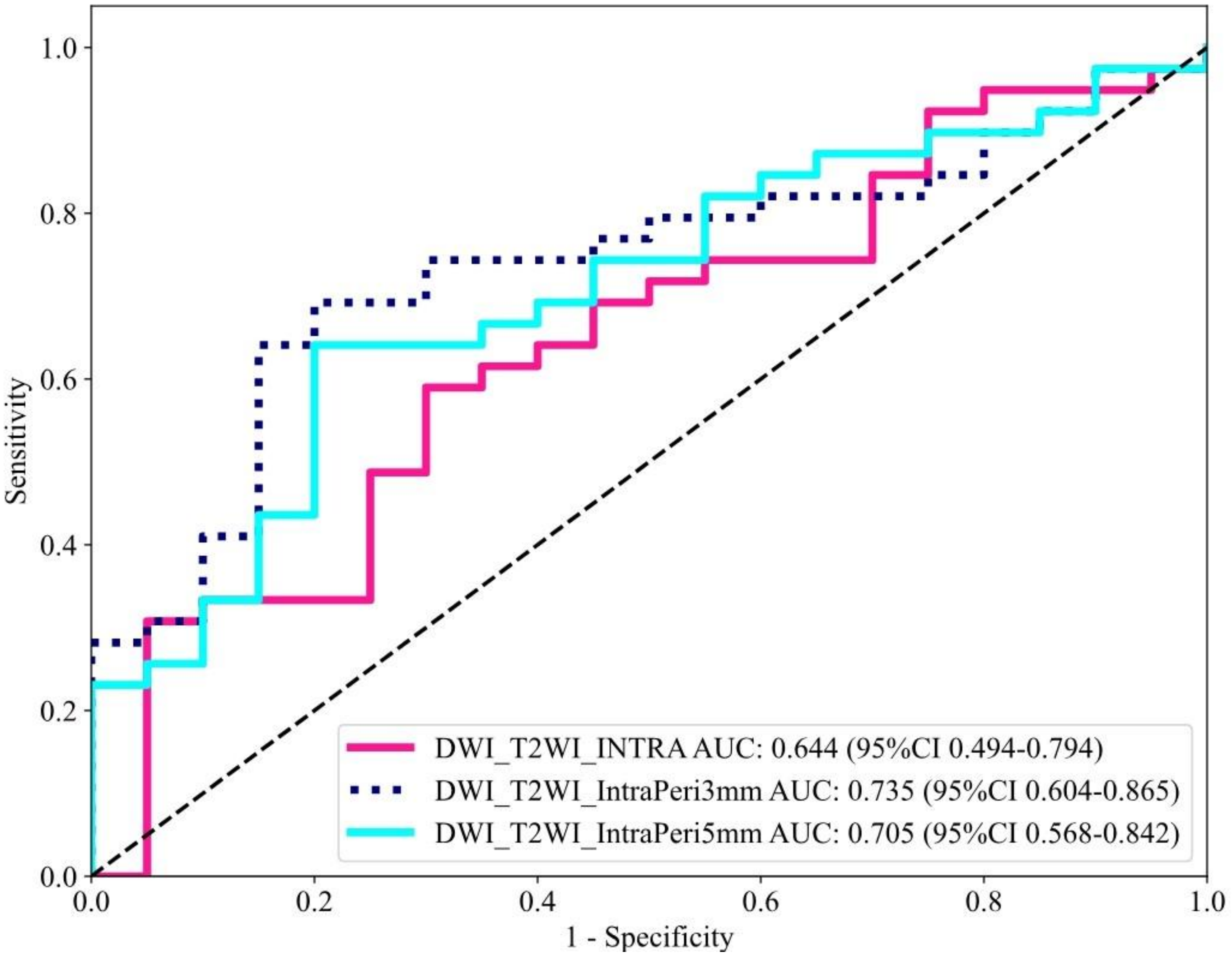

Sensitivity
1.0
0.8
0.6
0.4
0.2
0.0
0.0
0.2
0.4
0.6
0.8
1.0
1 - Specificity
DWI_T2WI_INTRA AUC: 0.644 (95%CI 0.494-0.794)
DWI_T2WI_IntraPeri3mm AUC: 0.735 (95%CI 0.604-0.865)
DWI_T2WI_IntraPeri5mm AUC: 0.705 (95%CI 0.568-0.842)

(b)

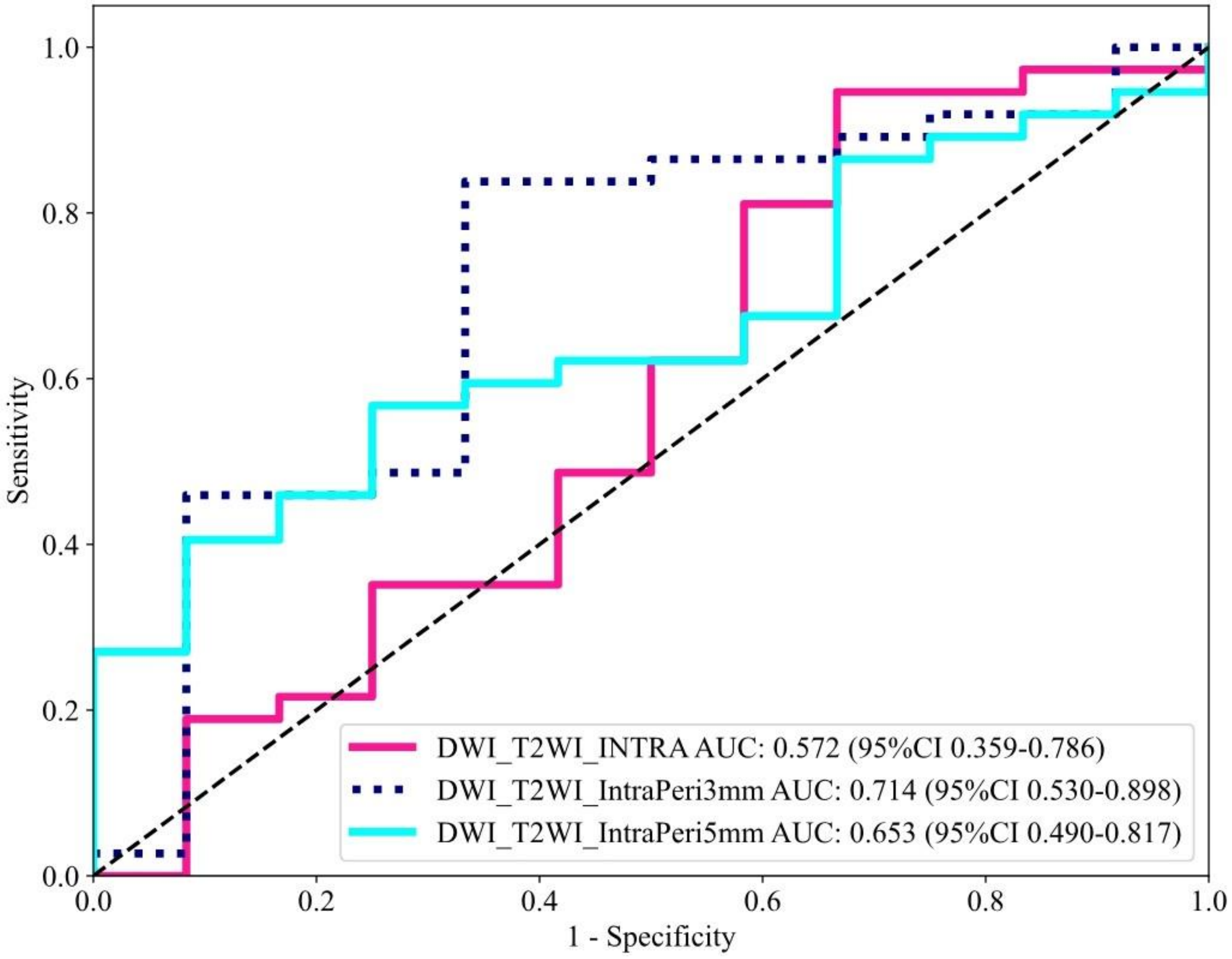

(c)

Fig.S3 ROC curves for training cohort (a), internal validation cohort (b), and external test cohort (c) of three models. "DWI_T2WI" denotes combined sequences. Model representations: pink solid line = DWI_T2WI_INTRA; dark blue dashed line = DWI_T2WI_IntraPeri3mm; light blue solid line = DWI_T2WI_IntraPeri5mm.

Table S4 The diagnostic performance of the DWI_T2WI_INTRA model, the DWI_T2WI_IntraPeri3mm model, and the DWI_T2WI_IntraPeri5mm model on the training, internal validation, and external test cohort for predicting LNM

Model	AUC	95%CI	*p*1	*p*2	Accuracy	Sensitivity	Specificity	PPV	NPV	Cohort
DWI_T2WI_INTRA	0.842	0.774-0.910			0.763	0.747	0.800	0.899	0.571	Train
DWI_T2WI_IntraPeri3mm	0.880	0.821-0.938	0.14		0.800	0.789	0.825	0.915	0.623	Train
DWI_T2WI_IntraPeri5mm	0.865	0.802-0.928	0.44	0.58	0.800	0.821	0.750	0.886	0.638	Train
DWI_T2WI_INTRA	0.644	0.494-0.794			0.627	0.590	0.700	0.793	0.467	Internal validation
DWI_T2WI_IntraPeri3mm	0.735	0.604-0.865	0.17		0.729	0.692	0.800	0.871	0.571	Internal validation
DWI_T2WI_IntraPeri5mm	0.705	0.568-0.842	0.31	0.49	0.695	0.641	0.800	0.862	0.533	Internal validation
DWI_T2WI_INTRA	0.572	0.359-0.786			0.796	0.946	0.333	0.814	0.667	External test
DWI_T2WI_IntraPeri3mm	0.714	0.530-0.898	0.04*		0.796	0.838	0.667	0.886	0.571	External test
DWI_T2WI_IntraPeri5mm	0.653	0.490-0.817	0.28	0.34	0.531	0.405	0.917	0.937	0.333	External test

*p*1: DWI_T2WI_INTRA compared with other models; *p*2: DWI_T2WI_IntraPeri3mm compared with DWI_T2WI_IntraPeri5mm, $p < 0.05$ indicates statistical significance
